# Trajectory Planning for a Spraying Robotic Arm Using a Digital Twin and an Improved SAC Algorithm

**DOI:** 10.3390/s26134285

**Published:** 2026-07-06

**Authors:** Bo Gao, Mingjun Xu, Liangsong Huang

**Affiliations:** Robot Research Center, Shandong University of Science and Technology, Qingdao 266590, China

**Keywords:** spraying robotic arm, digital twin, improved SAC algorithm, PER, composite reward function, trajectory planning

## Abstract

This paper proposes a trajectory planning method for a four-degree-of-freedom (4-DOF) spraying robotic arm based on a digital twin platform and an improved soft actor–critic (SAC) algorithm. The method addresses the complex environment of underground shotcrete spraying operations, the high cost and risk of physical robotic arm training, and the difficulty of incorporating spraying process constraints into traditional path planning methods. To enable unified modeling of the virtual prototype, operating scenario, reference trajectory, joint constraints, and spraying process proxy indicators, a digital twin platform for the spraying robotic arm was built using Unity Editor 2022.3.14f1c1.A composite reward function was designed to incorporate trajectory tracking, spray distance, nozzle normal, spraying speed, safety, and motion smoothness, and a prioritized experience replay (PER) mechanism based on double-critic temporal difference (TD) error was introduced to the conventional SAC algorithm. Simulation results show that, under the current digital twin environment and two-dimensional S-shaped reference trajectory, the Improved SAC algorithm reduces trajectory tracking root mean square error (RMSE) from 24.0 ± 2.0 mm to 8.0 ± 0.7 mm, corresponding to a 66.7% reduction compared with standard SAC. In addition, the spray distance error, nozzle normal error, spraying speed error, and motion smoothness index are reduced by 43.5%, 49.1%, 47.3%, and 48.7%, respectively.

## 1. Introduction

Underground shotcrete operations in mines are characterized by confined spaces, high dust concentrations, and complex working surfaces [1]. It is challenging to maintain uniformity in spray distance, nozzle orientation, and spraying speed over time when manual spraying or conventional teach-and-repeat methods are used [2]. This leads to high labor intensity and safety issues. Spraying robotic arms can improve the automation of spraying operations, but their trajectory planning must satisfy process constraints such as stable spray distance, nozzle normal alignment, uniform spraying speed, and smooth motion, in addition to accurate end-effector tracking of the reference path [3]. Complete six-degree-of-freedom (6-DOF) position tracking is challenging for 4-DOF spraying robotic arms due to their limited degrees of freedom. In order to better match the generated motion to realistic underground shotcrete scenarios, trajectory planning techniques should be developed taking into account the structural constraints and operating characteristics of spraying robotic arms.

Teach-and-repeat, geometric path planning, optimal control, and reinforcement learning are the main trajectory planning methods currently used for spraying and spray-painting robots [4]. Teach-and-repeat methods are easy to implement, but their adaptability is limited when the working surface geometry or spraying task changes. Geometric path planning methods can generate nozzle motion paths based on the geometry of known working surfaces, and point-cloud-based path planning methods are also effective for constructing spraying trajectories on complex surfaces [5]. However, these methods usually rely on accurate surface models and predefined trajectory structures. In addition, it remains difficult to simultaneously satisfy multiple spraying process constraints, including spray distance, nozzle normal error, spraying speed, and motion smoothness, during continuous trajectory execution. Spraying process constraints can be incorporated into the objective function using optimal control methods. However, in complex continuous-control tasks, optimal control methods usually require high model accuracy and high computational efficiency. Reinforcement learning, on the other hand, is suitable for multi-constraint trajectory planning because it can learn continuous-control policies through interaction with the environment [6].

Prioritized experience replay (PER) increases the use of high-value samples and has been used to improve the sampling efficiency of reinforcement learning [7]. Soft actor–critic (SAC) [8] is an off-policy continuous-control algorithm based on a maximum entropy framework with robust exploration capabilities. Nevertheless, directly applying standard SAC to trajectory planning for spraying robotic arms still presents two issues. First, a single trajectory-error reward cannot sufficiently capture spraying process requirements such as spray distance, nozzle normal error, spraying speed, safety constraints, and motion smoothness. Second, uniform experience replay struggles to emphasize critical samples, such as trajectory turning regions, abrupt spray distance changes, high-error regions, and states close to constraints. Therefore, it is necessary to adapt standard SAC to the trajectory planning task of spraying robotic arms by considering both spraying process constraints and sample utilization efficiency.

In order to provide a safe and repeatable training environment for reinforcement learning algorithms, digital twin technology makes it possible to create models in a virtual environment that match the physical structure of a robotic arm [9]. It also computes nozzle position, spray distance, normal vector, velocity, and collision status in real time [10]. It should be noted that the “Improved SAC” referred to in this paper does not propose a new theoretical framework for reinforcement learning, but rather represents an engineering adaptation of the standard SAC for the trajectory planning task of a 4-DOF spraying robotic arm. The following are this paper’s primary contributions:

1. A digital twin training environment is constructed for the spraying robotic arm. It integrates the robotic arm structural model, working surface, reference spraying trajectories, joint constraints, and spraying process proxy indicators, thereby providing a framework for state computation, action execution, and reward feedback in reinforcement learning.
2. A PER mechanism based on double-critic temporal difference (TD) error is introduced. This mechanism increases the sampling probability and utilization efficiency of critical samples, including trajectory turning regions, abrupt spray distance variations, and high-error regions.
3. A composite reward function is designed for the spraying robotic arm trajectory planning task. It incorporates nozzle position tracking, nozzle normal alignment, spray distance, spraying speed, control-amplitude, and motion smoothness. Simulation experiments are then conducted to verify the feasibility of the proposed method in the current digital twin environment.

## 2. Spraying Robotic Arm Modeling and Digital Twin Platform

### 2.1. Constructing a Digital Twin System

**Figure 1:**
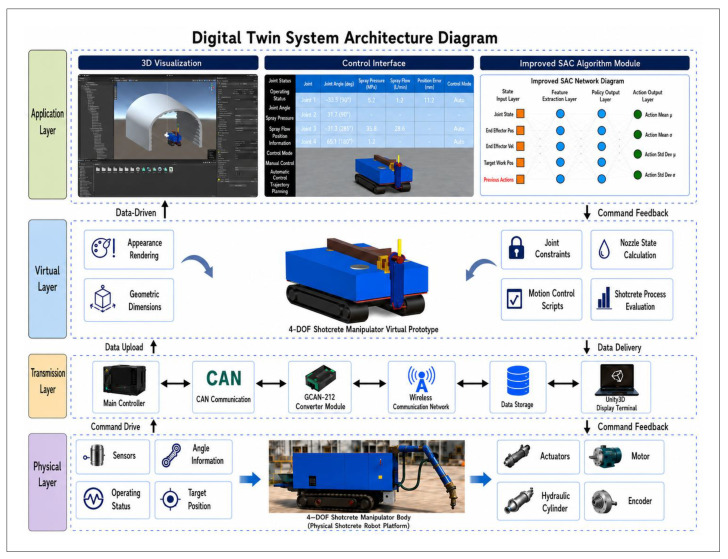
Four-layer architecture of the digital twin system for the spraying robotic arm.

In this study, a digital twin platform for a 4-DOF spraying robotic arm is constructed using Unity3D (version 1.9.3) [11]. The platform consists of the physical layer, transmission layer [12], virtual layer, and application layer. The physical layer includes the robotic arm, base controller, and joint status acquisition unit, which are used to obtain the telescopic arm displacement and the angles of the rotational joints [13]. Through a CAN bus, a GCAN-212 gateway, and network connectivity, the transmission layer enables data exchange between the physical prototype and the Unity host computer [14]. The virtual layer configures modules for joint constraints, motion limits, collision detection, and safety distance evaluation using a 3D model of the robotic arm to build a virtual prototype and a tunnel operation scenario. In order to carry out policy training, trajectory display, error statistics, and simulation verification, the application layer incorporates functions including trajectory planning, algorithm training, status monitoring, and result analysis. Figure 1 depicts the digital twin platform’s general architecture.

To ensure consistency between the virtual prototype and the physical robotic arm, this paper establishes a joint constraint model based on the Articulation Body component in Unity. The telescopic arm acts as the translational joint in the robotic arm’s “one translational and three rotational” serial structure, while the upper arm, forearm, and nozzle act as rotational joints. The rotational joints are configured as Revolute joints, and the telescopic joint is configured as a Prismatic joint. The joint variables, joint types, motion ranges, and velocity constraints are listed in Table 1.

The 4-DOF spraying robotic arm under consideration in this study is designed for underground shotcrete tunnel-like scenarios, where the spraying route is often arranged as a layered reciprocating trajectory and the working surface is mostly stretched along one direction. In contrast to a typical 6-DOF manipulator, the robotic arm under consideration is unable to independently control the nozzle’s entire position and orientation in any complex three-dimensional surface. Thus, nozzle position tracking, approximate normal direction alignment, spray distance maintenance, spraying speed stability, and motion smoothness are the primary goals of this study, which focuses on trajectory planning under limited shotcrete settings. This assumption is consistent with the two-dimensional S-shaped reference trajectory used in the current digital twin validation.

Basic tests were performed on the virtual–physical synchronization link to verify data exchange between the physical prototype and the Unity host computer. During the test, the spraying robotic arm continuously sent status signals to the host computer. The host computer recorded the number of abnormal messages, the message parsing accuracy, and the receiving interval between adjacent frames. According to the test results, the average refresh cycle for status data was 50.8 ms, with a minimum refresh interval of 49.1 ms and a maximum refresh interval of 78.6 ms. During the test, there were no error warnings and a 100% message parsing accuracy rate. In addition, bidirectional virtual–physical control tests showed that the status data from the physical prototype successfully drove synchronous updates in the Unity twin, and the target joint values generated in Unity were sent to the physical controller through the communication link. It should be mentioned that these tests cannot be directly compared with validation of long-term physical spraying operations because their main purpose was to confirm the communication link and joint-level interaction capabilities.

### 2.2. Kinematic Model of the Spraying Robotic Arm

Three rotational joints and one telescopic joint make up the spraying robotic arm. The definition of the joint variables at time *t* is as follows:

(1) $$q_{t}={\left[d_{t},\theta_{1,t},\theta_{2,t},\theta_{3,t}\right]}^{T}$$

The corresponding joint velocities are defined as follows:

(2) $${\dot{q}}_{t}={\left[{\dot{d}}_{t},{\dot{\theta }}_{1,t},{\dot{\theta }}_{2,t},{\dot{\theta }}_{3,t}\right]}^{T}$$

In the equation, $d_{t}$ denotes the displacement of the telescopic arm, while $\theta_{1,t}$, $\theta_{2,t}$, and $\theta_{3,t}$ denote the rotation angles of the upper arm, forearm, and nozzle, respectively.

According to the serial kinematic chain of the spraying robotic arm, the homogeneous transformation matrix of the nozzle tip with respect to the base coordinate system can be written as follows:

(3) $$T_{e}\left(q_{t}\right)=T_{d}\left(d_{t}\right)T_{1}\left(\theta_{1,t}\right)T_{2}\left(\theta_{2,t}\right)T_{3}\left(\theta_{3,t}\right)T_{tool}$$

The translational transformation matrix generated by the telescopic joint is denoted by $T_{d}\left(d_{t}\right)$. The pose transformation matrices generated by the rotational joints of the upper arm, forearm, and nozzle are denoted by $T_{1}\left(\theta_{1,t}\right)$, $T_{2}\left(\theta_{2,t}\right)$, and $T_{3}\left(\theta_{3,t}\right)$, respectively. The fixed transformation matrix from the tool joint coordinate system to the nozzle tip is denoted by $T_{tool}$. These transformation matrices are consistent with the coordinate definitions and mechanical dimensions of the Unity virtual prototype. Therefore, the detailed Denavit–Hartenberg (DH) parameters are not further expanded in this paper.

The homogeneous transformation matrix can be used to determine the nozzle tip’s position:

(4) $$p_{e,t}={\left[T_{e}\left(q_{t}\right)\right]}_{1:3,4}$$

where ${\left[T_{e}\left(q_{t}\right)\right]}_{1:3,4}$ represents the nozzle tip location in the base coordinate system, which is represented by the first three elements of the homogeneous transformation matrix’s fourth column.

The nozzle’s actual spray direction can be stated as follows:

(5) $$n_{spray,t}=R_{e}n_{0}$$

In this formula, $n_{0}$ is the unit vector of the spray direction in the nozzle coordinate system, and $R_{e}$ is the rotation matrix component in $T_{e}\left(q_{t}\right)$. In order to prevent confusion between the signs of the nozzle axis direction and the actual spray direction when defined in various coordinate systems, $n_{spray,t}$ is used instead of $n_{e,t}$.

In the digital twin environment, the joint states of the virtual robotic arm are updated according to the joint-velocity control commands. Let $u_{t}$ denote the actual joint-velocity command output by the agent at time *t*. The unconstrained joint variables are then calculated as follows:

(6) $${\tilde{q}}_{t+1}=q_{t}+u_{t}\Delta t$$

The joint variables are then constrained according to the joint limits:

(7) $$q_{t+1}=\mathrm{clip}\left({\tilde{q}}_{t+1},q_{\min},q_{\max}\right).$$

Here, clip(·) is applied component-wise to each joint variable to keep the joint variables within their allowable ranges. For a scalar variable *x* with lower bound *l* and upper bound *u*, the clipping operation is defined as

(8) clip(x,l,u)=min(max(x,l),u).

Therefore, for the *j*-th joint variable, Equation (7) can be written as

(9) $${\left[q_{t+1}\right]}_{j}=\min\left(\max\left({\left[{\tilde{q}}_{t+1}\right]}_{j},{\left[q_{\min}\right]}_{j}\right),{\left[q_{\max}\right]}_{j}\right),\;j=1,2,3,4.$$

In the above equations, Δt denotes the simulation control step size, while $q_{\min}$ and $q_{\max}$ denote the lower and upper bounds of the joint variables, respectively. Although the clipped joint variables are always kept within the feasible range, the out-of-bounds indicator is computed according to the unconstrained joint variables ${\tilde{q}}_{t+1}$. Specifically, $I_{\lim,t}=1$ if any component of ${\tilde{q}}_{t+1}$ exceeds its corresponding joint limit before clipping; otherwise, $I_{\lim,t}=0$. This treatment avoids the logical conflict between the joint limit penalty and the fact that the joint variables remain feasible after clipping. The indicator $I_{\lim,t}$ is subsequently used in the safety reward term to penalize infeasible actions.

### 2.3. Spraying Process Parameter Calculation

In this paper, a reference spraying trajectory is predefined on the working surface, and a spraying operation scenario is established in the digital twin environment [15]. A two-dimensional working surface trajectory is used to validate the algorithm in the simulation phase. The reference spraying trajectory points, the normal vector of the working surface, and the distance between the nozzle and the working surface are computed using the predefined model in the digital twin environment [16].

Let $p_{r}\left(\rho \right)$ be the reference spraying trajectory, where ρ∈[0,1] denotes the trajectory progress parameter. The unit tangent vector of the reference spraying trajectory is defined as

(10) $$\tau_{r}\left(\rho \right)=\frac{dp_{r}\left(\rho \right)/d\rho }{{\left\|dp_{r}\left(\rho \right)/d\rho \right\|}_{2}}$$

The unit normal vector of the working surface at pr(ρ) is denoted as $n_{r}\left(\rho \right)$, which is obtained from the predefined working surface model in the digital twin environment. The vector $n_{r}\left(\rho \right)$ is used later to calculate the nozzle normal error between the actual spray direction and the reference normal direction of the working surface.

The trajectory progress of the current nozzle tip is determined by finding the closest point on the reference spraying trajectory:

(11) $$\rho_{t}=\arg\min_{\rho }{∥p_{e,t}-p_{r}\left(\rho \right)∥}_{2}$$

The definition of the nozzle tip position error vector is as follows:

(12) $$e_{p,t}=p_{e,t}-p_{r}\left(\rho_{t}\right)$$

The definition of the position error scalar is as follows:

(13) $$E_{p,t}={∥e_{p,t}∥}_{2}$$

Specifically, state space construction uses $e_{p,t}$, but the reward function and RMSE evaluation utilize $E_{p,t}$. By doing this, the problem of inconsistent notation that would occur if position errors were utilized concurrently as scalars and vectors in various parts is avoided.

The angle between the nozzle’s actual spray direction and the working surface’s reference normal is known as the nozzle normal error:

(14) $$e_{n,t}=\arccos\left(\mathrm{clip}(n_{spray,t}^{T}n_{r}\left(\rho_{t}\right),-1,1)\right)$$

Both $n_{\mathrm{spray},t}$ and $n_{r}\left(\rho_{t}\right)$ are unit vectors. In theory, their inner product should lie within the range [−1,1]. However, small numerical errors may cause the value to slightly exceed this range during simulation. Therefore, the clip(·) operator is used to keep the input of the inverse cosine function within [−1,1].

The distance $d_{s,t}$ between the nozzle tip and the working surface is known as the spray distance.

The spray distance error is defined as follows:

(15) $$e_{d,t}=d_{s,t}-d_{ref}$$

The nozzle tip velocity is approximated as follows:

(16) $$v_{e,t}=\frac{p_{e,t}-p_{e,t-1}}{\Delta t}$$

Along the reference spraying trajectory, the nozzle’s tangential spraying speed is as follows:

(17) $$v_{s,t}=v_{e,t}^{T}\tau_{r}\left(\rho_{t}\right)$$

The spraying speed error is as follows:

(18) $$e_{v,t}=v_{s,t}-v_{ref}$$

This work defines a motion smoothness metric using normalized actions in order to avoid directly mixing translational and rotational velocity units [17]:

(19) $$S_{u}=\frac{1}{N-1}\sum_{t=2}^{N}{∥{\bar{u}}_{t}-{\bar{u}}_{t-1}∥}_{2}^{2}$$

Here, ${\bar{u}}_{t}$ denotes the normalized action output from the actor network at time step *t*, and *N* denotes the number of time steps in one evaluation trajectory. Since the robotic arm contains both a translational joint and rotational joints, directly calculating smoothness using the actual joint-velocity command $u_{t}$ would mix different physical units. Therefore, the normalized action ${\bar{u}}_{t}$ is used to evaluate the continuity of the control command in a dimensionless form. This smoothness metric is used as an evaluation indicator, and the same normalized action difference is also introduced into the composite reward function to penalize abrupt changes in the control command. It should be noted that actual spray quality metrics, such as coating-thickness uniformity, rebound rate, and construction efficiency, cannot yet be directly equated with the spray distance, nozzle normal error, spraying speed, and motion smoothness discussed in this paper, because these metrics are only spraying process proxy indicators.

## 3. Improvements to the Design of the SAC Algorithm

### 3.1. Markov Decision Process Modeling

The agent outputs joint-velocity commands instead of joint torques because the spraying robotic arm is in joint-velocity control mode. As a result, the forward kinematics from joint space to nozzle position and spray direction, as well as the kinematic update of the joint variables, are the primary determinants of the state transition in the current digital twin environment. For this reason, this study uses a kinematic model. The lack of specific modeling of dynamic effects, actuator delays, hydraulic response characteristics, and long-term physical spraying disturbances is regarded as a constraint for further physical validation.

Based on the kinematic model of the spraying robotic arm established in Section 2.2 and the spraying process proxy indicators defined in Section 2.3, this paper models the trajectory planning problem as a Markov decision process with a continuous state space and a continuous action space [18]:

(20) M=(S,A,P,R,γ)

The state space is denoted by S, and the action space by *A*, the state transition probability by *P*, the instantaneous reward function by *R*, and the discount factor by γ in the equation.

This work specifies the state at time t as follows so that the agent can simultaneously perceive the motion state of the robotic arm, spraying process deviations, and trajectory progress [19]:

(21) $$s_{t}={\left[q_{t},{\dot{q}}_{t},{\bar{u}}_{t-1},e_{p,t},e_{n,t},e_{d,t},e_{v,t},\rho_{t}\right]}^{T}$$

The 4-dimensional joint variables are denoted by $q_{t}$, the 4-dimensional joint velocities by ${\dot{q}}_{t}$, the 4-dimensional normalized motion at the previous time step by ${\bar{u}}_{t-1}$, the 3-dimensional position error vector by $e_{p,t}$, and the scalars $e_{n,t}$, $e_{d,t}$, $e_{v,t}$, and $\rho_{t}$. As a result, this paper’s state space has 19 dimensions. The goal of incorporating the motion from the previous time step into the state space is to satisfy the Markovian property by ensuring that the motion smoothness penalty is determined jointly by the current state and the current action.

The action of the agent is defined as the joint-velocity command of the four joints:

(22) $$u_{t}={\left[{\dot{d}}_{t},{\dot{\theta }}_{1,t},{\dot{\theta }}_{2,t},{\dot{\theta }}_{3,t}\right]}^{T}$$

The actor network outputs a normalized action:

(23) $${\bar{u}}_{t}\in {\left[-1,1\right]}^{4}$$

The actual joint-velocity command is obtained by scaling the normalized action:

(24) $$u_{t}={\bar{u}}_{t}⊙u_{\max}$$

In Equation (22), $u_{\max}$ represents the velocity limit vector of the four joints, and ⊙ denotes element-wise multiplication. The specific values of $u_{\max}$ are consistent with the velocity constraints listed in Table 1 and the algorithm parameters listed in Table 2. In the current prototype, the upper and lower velocity limits of each joint are symmetric; therefore, the normalized action ${\bar{u}}_{t}\in {\left[-1,1\right]}^{4}$ output by the actor network can be directly scaled by $u_{\max}$ to obtain the actual joint-velocity command $u_{t}$. Through this scaling process, the actor output is constrained by the actuator velocity limits before being applied to the digital twin environment.

After the velocity command $u_{t}$ is executed, the unconstrained joint variable ${\tilde{q}}_{t+1}$ is first calculated according to the kinematic update, and then the joint-position limits are enforced using the clipping operation defined in Section 2.2. If any component of ${\tilde{q}}_{t+1}$ exceeds its allowable range before clipping, the out-of-bounds indicator $I_{\lim,t}$ is activated and a penalty is introduced through the safety reward term. Therefore, the joint constraints are involved not only in action execution but also in state transition and reward feedback during policy training. If a future physical prototype uses asymmetric actuator velocity limits, the motion scaling relationship should be adjusted according to the actual lower and upper velocity limits.

### 3.2. Fundamentals of the Standard SAC Algorithm

Designed for continuous action spaces, SAC is a maximum entropy reinforcement learning algorithm. Its goal is to optimize cumulative reward while improving policy exploration capabilities by adding a policy entropy term:

(25) $$J\left(\pi \right)=E_{\pi }\left[\sum_{t=0}^{T}\gamma^{t}\left(r_{t}+\alpha H\left(\pi \left(\cdot |s_{t}\right)\right)\right)\right]$$

The temperature coefficient, represented by α in the equation, is used to modify the relative weights of the reward and entropy terms [20]. For all comparison algorithms in this paper, α is fixed at 0.20. This parameter does not improve the algorithm presented in this paper; rather, it is utilized only to guarantee consistency in the ablation experiments. Future studies will compare the adaptive temperature control version in more detail.

A double-critic network is used by the SAC method to alleviate the overestimation of Q-values. To avoid confusion with the joint-angle variables $\theta_{1,t}$, $\theta_{2,t}$, and $\theta_{3,t}$ defined in Section 2.2, the parameters of the two critic networks are denoted by $\psi_{1}$ and $\psi_{2}$, respectively. The two critic networks are represented as

(26) $$Q_{\psi_{1}}\left(s_{t},u_{t}\right),\;Q_{\psi_{2}}\left(s_{t},u_{t}\right).$$

The corresponding target critic networks are represented as

(27) $$Q_{{\bar{\psi }}_{1}}\left(s_{t},u_{t}\right),\;Q_{{\bar{\psi }}_{2}}\left(s_{t},u_{t}\right).$$

The soft-updating technique is employed by the target critic networks:

$${\bar{\psi }}_{i}\leftarrow \tau \psi_{i}+\left(1-\tau \right){\bar{\psi }}_{i},\;i=1,2.$$

For the standard SAC, the soft Bellman target is

(28) $$y_{t}=r_{t}+\gamma \left(1-D_{t}\right)\left[\min_{i=1,2}Q_{{\bar{\psi }}_{i}}\left(s_{t+1},u_{t+1}\right)-\alpha \log\pi_{\phi }\left({\bar{u}}_{t+1}|s_{t+1}\right)\right].$$

In this case, the termination flag is indicated by $D_{t}$. $D_{t}=1$ and no additional state-value estimate is bootstrapped when the robotic arm collides, surpasses a joint limit, finishes the current trajectory, or reaches the maximum step size.

The critic network loss function is

(29) $$J_{Q}\left(\psi_{i}\right)=\frac{1}{2}E\left[{\left(Q_{\psi_{i}}\left(s_{t},u_{t}\right)-y_{t}\right)}^{2}\right].$$

The actor network loss function is

(30) $$J_{\pi }\left(\phi \right)=E\left[\alpha \log\pi_{\phi }\left({\bar{u}}_{t}|s_{t}\right)-\min_{i=1,2}Q_{\psi_{i}}\left(s_{t},u_{t}\right)\right].$$

### 3.3. PER Based on Double-Critic TD Error

Highlighting important samples is challenging since standard SAC uses uniform experience replay. In this paper, a double-critic TD error-based PER system is presented. The *i*-th sample in the replay buffer is denoted as

(31) $$\xi_{i}=\left(s_{i},u_{i},r_{i},s_{i}^{\prime },D_{i}\right).$$

For the two critic networks, the temporal difference (TD) errors are

(32) $$\delta_{k,i}=y_{i}-Q_{\psi_{k}}\left(s_{i},u_{i}\right),\;k=1,2.$$

The sample priority is defined as

(33) $$p_{i}=\frac{|\delta_{1,i}\left|+\right|\delta_{2,i}|}{2}+\varepsilon .$$

The small constant ε prevents samples with zero TD error from being entirely ignored. A sample’s sampling probability is

(34) $$P\left(i\right)=\frac{p_{i}^{\eta }}{\sum_{j}p_{j}^{\eta }}.$$

The priority exponent is denoted by η. When η=0, the sampling strategy becomes uniform random sampling. Importance sampling weights are introduced to reduce the distribution bias caused by prioritized sampling:

(35) $$w_{i}=\frac{{\left(NP\left(i\right)\right)}^{-\beta }}{\max_{j}{\left(NP\left(j\right)\right)}^{-\beta }}.$$

The critic network loss function with importance sampling weights can be written as

(36) $$J_{Q}^{\mathrm{PER}}\left(\psi_{k}\right)=E\left[w_{i}{\left(Q_{\psi_{k}}\left(s_{i},u_{i}\right)-y_{i}\right)}^{2}\right],\;k=1,2.$$

From the perspective of dynamic programming, the temporal difference (TD) error can be interpreted as a sample-based Bellman residual. In the SAC framework, the soft Bellman target $y_{i}$ provides a one-step estimate of the expected soft return, while $Q_{\psi_{k}}\left(s_{i},u_{i}\right)$ is the current value estimated by the *k*-th critic network. Therefore, the TD error is defined as

(37) $$\delta_{k,i}=y_{i}-Q_{\psi_{k}}\left(s_{i},u_{i}\right).$$

It measures the degree to which the current critic network violates the soft Bellman consistency condition at sample $\xi_{i}$. Minimizing the critic loss is therefore equivalent to reducing the squared soft Bellman residual over sampled transitions.

For continuous-control trajectory planning, the Hamilton–Jacobi–Bellman (HJB) equation gives the optimality condition of the value function in continuous-time optimal control. The Bellman consistency relation used in SAC can be regarded as its discrete-time, sample-based counterpart under the Markov decision process formulation. In this study, the HJB equation is not solved explicitly. Instead, the critic networks approximate the soft action-value function by minimizing the empirical Bellman residual. Samples with larger TD errors correspond to larger Bellman residuals and are therefore assigned higher priorities in PER. This allows the policy update to focus more on trajectory transition regions, high-error states, and constraint-sensitive states during training.

By increasing the utilization rate of samples in high-error zones, trajectory transition zones, spray distance deviation regions, and areas close to obstacles, this technique increases training convergence performance and sample utilization efficiency.

### 3.4. Design of the Composite Reward Function

This study develops a composite reward function that incorporates spraying process constraints and motion smoothness in order to concurrently satisfy multiple objectives, including nozzle trajectory tracking, normal direction alignment, spray distance maintenance, spraying speed stability, and motion smoothness [21]. The total reward at time step *t* is defined as

(38) $$r_{t}=r_{\mathrm{tr}}\left(t\right)+r_{\mathrm{pro}}\left(t\right)+r_{u}\left(t\right)+r_{\mathrm{safe}}\left(t\right)+r_{\mathrm{goal}}\left(t\right)+r_{\Delta u}\left(t\right).$$

The trajectory tracking reward is defined as

(39) $$r_{\mathrm{tr}}\left(t\right)=-w_{p}E_{p,t}^{2}-w_{n}e_{n,t}^{2}.$$

In this case, $e_{n,t}$ represents the nozzle normal error and $E_{p,t}$ represents the scalar position error between the nozzle tip and the reference spraying trajectory. Positive weighting coefficients $w_{p}$ and $w_{n}$ are utilized to modify the relative significance of nozzle normal direction alignment and position tracking precision, respectively.

The spraying process constraint reward is defined as

(40) $$r_{\mathrm{pro}}\left(t\right)=-w_{d}e_{d,t}^{2}-w_{v}e_{v,t}^{2}.$$

In this case, the spray distance error is denoted by $e_{d,t}$ and the spraying speed error by $e_{v,t}$. The spray distance limitation and the spraying speed restriction are balanced by the positive weighting coefficients $w_{d}$ and $w_{v}$.

The control-amplitude penalty term is defined as

(41) $$r_{u}\left(t\right)=-w_{u}{\left|{\bar{u}}_{t}\right|}_{2}^{2}.$$

In this case, $w_{u}$ is the control-amplitude penalty coefficient, and ${\bar{u}}_{t}$ represents the normalized action produced by the actor network. Because the motion described in this study is controlled by joint-velocity commands rather than joint torques, this term is called a control-amplitude penalty instead of an energy penalty. Using the normalized action ${\bar{u}}_{t}$ also avoids directly summing joint velocities with different physical units.

The safety constraint reward is defined as

(42) $$r_{\mathrm{safe}}\left(t\right)=-C_{\mathrm{col}}I_{\mathrm{col}}-C_{\lim}I_{\lim}-w_{o}{\left[\max(0,d_{\mathrm{safe}}-d_{\min,t})\right]}^{2}.$$

In this case, the collision indicator function, $I_{\mathrm{col}}$, is 0 in the absence of a collision and 1 in the event of one. The joint out-of-bounds indicator function, $I_{\lim}$, has a value of 1 when an unconstrained joint variable surpasses its permitted limit and 0 otherwise. The robotic arm’s minimal distance from the obstruction is represented by the variable $d_{\min,t}$, while the safety distance threshold is represented by $d_{\mathrm{safe}}$. The weighting coefficient for the obstacle-distance penalty is $w_{o}$, while the penalty coefficients for collision and joint limit violation are $C_{\mathrm{col}}$ and $C_{\lim}$, respectively.

The trajectory-completion reward is defined as

(43) $$r_{\mathrm{goal}}\left(t\right)=w_{g}I_{\mathrm{goal}}\left(t\right).$$

In this case, the trajectory-completion indicator function, denoted by $I_{\mathrm{goal}}\left(t\right)$, has a value of 1 when the agent finishes the reference spraying trajectory and 0 otherwise. The positive reward coefficient for completing a trajectory successfully is represented by the parameter $w_{g}$.

The motion smoothness penalty term is defined as

(44) $$r_{\Delta u}\left(t\right)=-w_{\Delta u}{∥{\bar{u}}_{t}-{\bar{u}}_{t-1}∥}_{2}^{2}.$$

In this case, the action-variation penalty’s weighting coefficient is denoted by $w_{\Delta u}$. This term penalizes abrupt changes in the normalized action and encourages smoother joint-velocity commands. Instead of being added separately to the actor loss function, the motion smoothness constraint is incorporated into the composite reward function to avoid redundant penalties.

The composite reward function’s weighting coefficients are all positive hyperparameters. The relative relevance of trajectory tracking accuracy, spraying process constraints, control-amplitude limitation, safety, trajectory-completion, and motion smoothness is determined by the parameters $w_{p}$, $w_{n}$, $w_{d}$, $w_{v}$, $w_{u}$, $w_{o}$, $w_{g}$, and $w_{\Delta u}$. Stronger penalties are applied for collision and joint limit violation occurrences using the penalty coefficients $C_{\mathrm{col}}$ and $C_{\lim}$. The detailed numerical values of these parameters are listed in Table 2.

## 4. Improved SAC Training Procedure in a Digital Twin Environment

**Figure 2:**
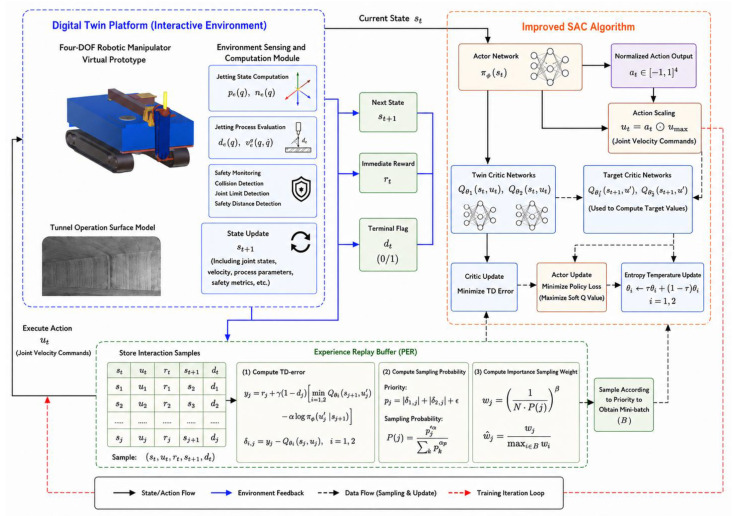
Closed-loop interaction between the Improved SAC algorithm and the digital twin platform. The blue dashed box represents the digital twin platform, the orange dashed box represents the Improved SAC algorithm, and the green dashed box represents the experience replay buffer with prioritized experience replay. The green feedback boxes between the digital twin platform and the algorithm indicate the next state, immediate reward, and terminal flag. The blue and orange boxes inside the Improved SAC algorithm represent critic-related and actor/action-related modules, respectively. The arrows indicate state/action flow, environment feedback, data flow for sampling and updating, and the training iteration loop.

In order to elucidate the coupling relationship between the Improved SAC algorithm and the digital twin platform, this study develops a closed-loop training procedure that includes “state awareness—action execution—reward feedback—sample storage—policy update.” The digital twin platform continuously provides feedback on the robotic arm’s state, the nozzle’s spatial position, spray distance, nozzle normal, spraying speed, collision status, and safety distance. This information is then translated into state variables, reward values, and termination conditions. This process goes beyond simply running the SAC algorithm in a simulation environment. Figure 2 depicts the closed-loop interaction process between the Improved SAC algorithm and the digital twin platform.

The robotic arm’s joint variables, joint velocities, reference spraying trajectory, working surface model, target spray distance, target spraying speed, and safety distance threshold are all initialized by the digital twin platform at the beginning of training. The platform then uses the current joint states to compute the nozzle tip location, spray direction, spray distance error, normal error, spraying speed error, and trajectory progress. The agent’s state is created by combining this data with the normalized motion from the preceding time step. This state allows the agent to make integrated decisions between trajectory tracking and spraying process constraints by reflecting both the robotic arm’s present motion and the nozzle’s process deviation with respect to the working surface.

In the reward feedback phase, the digital twin platform generates an instantaneous reward based on a composite reward function while concurrently calculating trajectory tracking error, spray distance error, nozzle normal error, spraying speed error, safety status, and motion changes. The nozzle is guided toward the reference spraying trajectory by the trajectory tracking term; the relative relationship between the nozzle and the working surface is constrained by the spray distance, normal, and speed terms; collisions and boundary violations are suppressed by the safety term; and sudden changes in control commands are lessened by the motion smoothing term. A sample $\left(s_{t},u_{t},r_{t},s_{t+1},D_{t}\right)$ is produced as a result and is kept in the replay buffer.

During the policy updating phase, the algorithm determines sample priority based on the TD errors of the two critic networks. Samples with higher TD errors are more likely to be selected in subsequent mini-batch sampling because they often correspond to critical stages, such as trajectory turning regions, abrupt spray distance changes, high-error regions, or proximity to obstacles. An importance sampling weight is added to the critic loss function in order to reduce the distribution bias caused by prioritized sampling. The actor network then updates the policy parameters based on policy entropy and soft Q-values, while the critic network updates the Q-function based on the weighted loss. Through soft updates, the target critic network preserves training stability. The priority of the related samples in the replay buffer is updated based on the new TD error following each network update. Until the policy converges or the maximum number of training episodes is reached, this procedure is repeated.

Through this closed-loop process, the digital twin platform participates in state computation, motion execution, process parameter feedback, safety evaluation, and sample generation, in addition to 3D visualization and trajectory display. The Improved SAC algorithm continuously optimizes the joint-velocity control strategy based on platform feedback. Together, these elements form a simulation training system for trajectory planning of spraying robotic arms and provide the basis for convergence analysis, ablation experiments, trajectory tracking verification, and evaluation of spraying process proxy indicators.

## 5. Simulation Validation and Results Analysis

### 5.1. Experimental Setup

In actual spraying operations, the spraying robot usually follows a layered reciprocating motion strategy. The planned trajectory is composed of multiple horizontal scanning lines and vertical transition segments. During operation, the nozzle remains approximately perpendicular to the wall surface and moves sequentially from top to bottom along the predefined path. The horizontal scanning trajectories are used to cover the target spraying area, while adjacent trajectories are connected through end-transition segments, thereby forming an S-shaped reciprocating path.

This study performs four sets of comparative experiments—standard SAC, SAC+PER, SAC+Composite Reward, and Improved SAC—on the same digital twin platform to confirm the efficacy of the Improved SAC algorithm in the trajectory planning task for a spraying robotic arm. This research uses eight predefined random seeds for independent training, specified as 2026, 2027, 2028, 2029, 2030, 2031, 2032, and 2033, to reduce the influence of random initialization and exploration noise on the experimental outcomes. The mean and standard deviation of the outcomes from the eight training runs are computed, and all comparison algorithms use the same set of random seeds.

A two-dimensional reference spraying trajectory with 420 sampling points is used for trajectory tracking validation. This trajectory is suitable for verifying the algorithm’s tracking performance on nonlinear trajectories because it contains segments with gradual changes and local curvature variations. Table 2 summarizes the experimental setup, ablation setups, key SAC parameters, PER parameters, reward function parameters, and evaluation settings to enhance the reproducibility and clarity of the simulation studies.

This research performs trials under the same settings for the digital twin environment, reference spraying trajectory, number of training iterations, motion limits, and random seeds to ensure reproducibility and fairness among the compared methods. The network architecture, basic training parameters, simulation step size, and motion constraints are the same for all compared methods, except for the introduction of PER and the composite reward function. Nozzle tip trajectory planning accuracy is evaluated using trajectory tracking RMSE as the primary metric [22]. Spraying process proxy indicators and motion smoothness are evaluated using secondary metrics, including spray distance error, nozzle normal error, spraying speed error, and the motion smoothness index. After independent training, all metrics are calculated during the evaluation phase, and lower values indicate better performance. The statistical unit is the number of independent training runs rather than the 420 trajectory sampling points. The 420 sampling points are not regarded as independent statistical samples; they are only used to calculate the error of a single evaluation trajectory.

### 5.2. Comparison of Algorithm Convergence Performance

Because different algorithms employ distinct reward functions, reward scales may affect a direct comparison of raw cumulative rewards. Therefore, convergence performance is assessed using normalized evaluation scores. The raw episodic evaluation score of algorithm *a* under random seed *s* at episode *e* is represented by $R_{a,s}\left(e\right)$. To reduce high-frequency fluctuations, a moving-average smoothing technique with a window size of nine is first used. Next, the normalized evaluation score is computed as

(45) $$S_{a,s}\left(e\right)=\frac{{\bar{R}}_{a,s}\left(e\right)-R_{\min}}{R_{\max}-R_{\min}+\epsilon_{n}}$$

where $R_{\min}$ and $R_{\max}$ represent the lowest and highest values of the smoothed scores among all compared algorithms, random seeds, and training episodes, and ${\bar{R}}_{a,s}\left(e\right)$ represents the smoothed episodic evaluation score. To prevent division by zero, the small constant $\epsilon_{n}=1.0\times 10^{-8}$ is employed. Better convergence performance is indicated by a higher normalized evaluation score.

**Figure 3:**
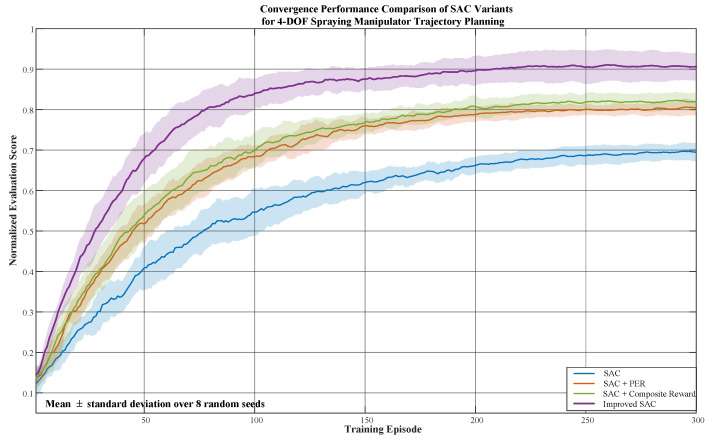
Comparison of convergence performance among different algorithms. The curves represent the mean normalized evaluation scores over eight random seeds, and the shaded regions denote the corresponding standard deviations. A moving-average smoothing window of nine is used for visualization.

Figure 3 compares the convergence performance of the different comparison methods with the Improved SAC algorithm. The solid line represents the mean normalized evaluation score from eight independent training runs, while the shaded region indicates the corresponding standard deviation range.

As shown in Figure 3, the standard SAC method converges slowly and yields the lowest final normalized evaluation score. SAC+PER exhibits a quicker rise in the normalized score with the introduction of PER, suggesting that the PER approach enhances the use of important transition samples. The resulting normalized evaluation score of SAC+Composite Reward is greater than that of standard SAC SAC, indicating that spraying process proxy constraints and motion smoothness constraints provide more effective policy guidance. The quick score increase of Improved SAC in the early training phase and its high normalized evaluation score in the later training phase demonstrate that the two improvements play complementary roles in the current simulation task. It should be noted that the main purpose of Figure 3 is to show training trends; the evaluation phase’s trajectory errors and spraying process proxy indicators continue to provide the foundation for quantitative performance analysis.

Several diagnostic indicators, including the episodic reward, the episodic cost, and the average absolute TD error, were also monitored during training in order to better investigate the training process without introducing additional figures. These indicators are defined as

(46) $$\begin{array}{cc} R_{e} & =\sum_{t=1}^{T_{e}}r_{t},\;C_{e}=\sum_{t=1}^{T_{e}}c_{t},\;{\bar{\delta }}_{\mathrm{TD}}\left(e\right)=\frac{1}{2M_{e}}\sum_{i=1}^{M_{e}}\left(|\delta_{1,i}\left|+\right|\delta_{2,i}|\right). \end{array}$$

Here, $R_{e}$ denotes the cumulative reward of episode *e*, $C_{e}$ denotes the accumulated positive cost corresponding to the tracking, spraying process, smoothness, and safety-related penalty terms defined in the composite reward function, and $T_{e}$ denotes the number of time steps in episode *e*. The variable $c_{t}$ represents the positive penalty cost at time step *t*. $M_{e}$ denotes the number of sampled transitions used for critic updates in episode *e*, while $\delta_{1,i}$ and $\delta_{2,i}$ are the TD errors of the two critic networks. During training, $R_{e}$ is expected to increase and gradually stabilize, whereas $C_{e}$ and ${\bar{\delta }}_{\mathrm{TD}}\left(e\right)$ are expected to decrease and fluctuate within a limited range after convergence. These diagnostic indicators provide additional training process evidence for reward accumulation, constraint-cost reduction, and critic fitting stability, which is consistent with the convergence trend shown in Figure 3.

### 5.3. Ablation Study

**Figure 4:**
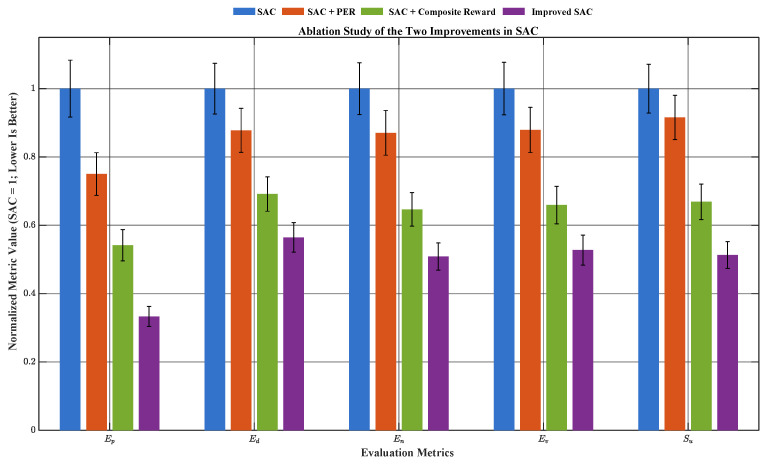
Ablation analysis of the proposed SAC improvements. All indicators are normalized relative to the standard SAC method, where SAC = 1. Smaller values indicate better performance.

This paper performs an ablation analysis using trajectory tracking RMSE as the primary evaluation metric, with spray distance error, nozzle normal error, spraying speed error, and the motion smoothness index serving as secondary evaluation metrics, in order to confirm the efficacy of the two improvements—PER and the composite reward function. Figure 4 displays the ablation results for the two improvements, and Table 3 provides the detailed performance statistics.

Since all indicators in Figure 4 are normalized relative to the standard SAC results, where SAC = 1, a lower value denotes better performance. As shown in Figure 4 and Table 3, the trajectory tracking RMSE of the standard SAC is 24.0±2.0mm. This value is reduced to 18.0±1.5mm for SAC+PER, 13.0±1.1mm for SAC+Composite Reward, and 8.0±0.7mm for Improved SAC. Based on the mean and standard deviation of eight independent training runs, the 95% t-confidence interval for the mean RMSE of standard SAC is 22.33–25.67 mm, whereas that of Improved SAC is 7.41–8.59 mm. The descriptive statistics show that Improved SAC reduces the trajectory tracking RMSE by 66.7% compared with standard SAC, indicating that the proposed improvements significantly enhance the trajectory tracking accuracy of the spraying robotic arm.

Improved SAC attains a spray distance error of 23.6±1.8mm, a nozzle normal error of $5.9\pm 0.46^{\circ }$, a spraying speed error of 0.048±0.004m/s, and a motion smoothness index of 0.079±0.006 for the secondary evaluation metrics. These four indices are 43.5%, 49.1%, 47.3%, and 48.7% lower than those of standard SAC, respectively. These findings demonstrate that the proposed method improves trajectory tracking accuracy, spraying process proxy indicators, and joint-velocity command smoothness.

The increased use of informative samples is the primary source of SAC+PER’s improvement. Such samples frequently exist in trajectory turning areas, high-error states, regions with significant spray distance variability, and states near motion or safety limits in the spraying trajectory planning task. PER allows the policy and the critic networks to learn from challenging trajectory segments more frequently by giving higher sampling probability to transitions with larger TD errors. As a result, SAC+PER lowers the trajectory tracking RMSE, but because the reward structure is not altered, its improvement in metrics relating to the spraying process is somewhat limited.

SAC+Composite Reward shows more evident reductions in spray distance error, nozzle normal error, spraying speed error, and the motion smoothness index. This is because the composite reward function explicitly incorporates trajectory tracking, spray distance maintenance, nozzle normal alignment, spraying speed stability, safety constraints, and motion smoothness into the policy optimization objective. Therefore, the learned policy is more consistent with the actual requirements of the spraying task. The Improved SAC obtains the lowest mean values across all five metrics, indicating that PER and the composite reward function have complementary effects. Specifically, the composite reward function determines what the agent should optimize, while PER improves which samples are emphasized during training. Therefore, the ablation results verify that the performance gain of Improved SAC is produced by the joint effect of task-oriented reward design and prioritized sample utilization.

### 5.4. Accuracy of Trajectory Tracking Verification

**Figure 5:**
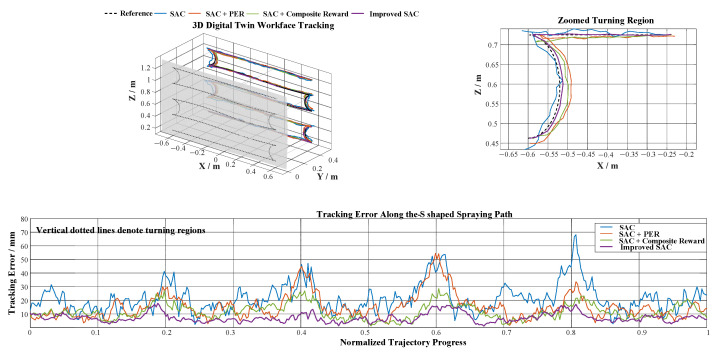
Trajectory tracking performance comparison of different algorithms.

The difference between the actual nozzle trajectory and the reference spraying trajectory is quantitatively evaluated using the root mean square error (RMSE), which serves as the main evaluation metric in this work. The nozzle tip is closer to the predetermined spraying path when the RMSE is smaller. To further assess the effectiveness of different algorithms in the reference spraying trajectory tracking task, this study uses a two-dimensional S-shaped reference spraying trajectory on a working surface within a 3D digital twin environment. This trajectory reflects common features of multi-pass spraying operations performed by spraying robotic arms and consists of several lateral spraying segments and line-change turning segments [23]. Figure 5 shows the trajectory tracking accuracy validation results for the compared algorithms.

The working surface is represented by the gray surface in Figure 5, the reference nozzle trajectory produced while maintaining the intended spray distance is represented by the black dashed line, and the actual nozzle trajectories produced by each algorithm are represented by the curves of various colors. The spatial relationship between the reference spraying trajectory and the actual trajectory close to the digital twin working surface is depicted in the 3D view. Tracking errors at the line-change and turning zones may be seen in the zoomed-in portion, and the error curve at the bottom shows how tracking errors vary along the normalized trajectory. It should be mentioned that this experiment cannot yet be compared to a full spraying validation on intricate 3D tunnel surfaces because it is still a 2D working surface trajectory validation within a 3D digital twin environment.

As shown in Figure 5, when the nozzle shifts from a lateral spraying segment to a line-change turning region, it is difficult to coordinate position adjustment and velocity switching. The standard SAC therefore shows a relatively prominent tracking error peak near the line-change turning region. Prioritizing experience replay may enhance the use of samples in the line-change turning region and high-error locations, as indicated by the somewhat lower error peak of SAC+PER. Constraints on spray distance, normal direction, velocity, and motion smoothness can enhance trajectory execution stability, as evidenced by the SAC+composite reward function’s real trajectory getting closer to the reference spraying trajectory. With comparatively reduced error variations along the trajectory, the Improved SAC, which concurrently integrates PER and a composite reward function, generates the trajectory that is closest to the reference spraying trajectory. Table 3 illustrates how the RMSE of the trajectory for the Improved SAC dropped by 66.7%, from 24.0 ± 2.0 mm for the standard SAC to 8.0 ± 0.7 mm. These findings show that the proposed method can improve the nozzle trajectory tracking accuracy of a 4-DOF spraying robotic arm under the existing digital twin environment and two-dimensional S-shaped reference spraying trajectory conditions.

### 5.5. Validation of Motion Smoothness and Spraying Process Constraints

**Figure 6:**
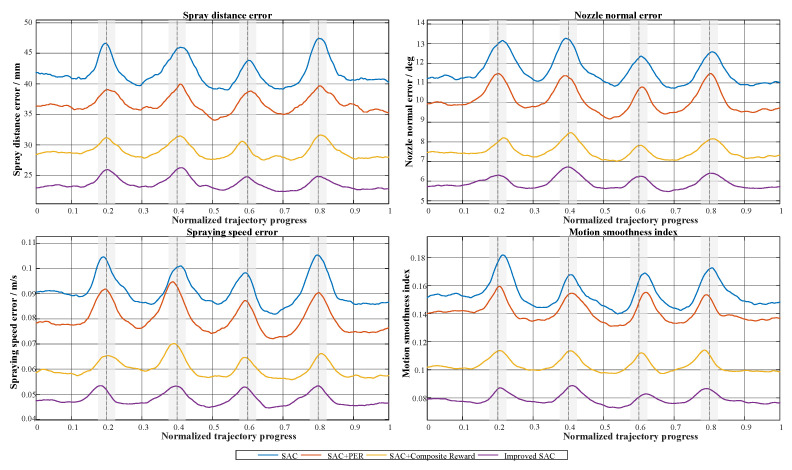
Comparison of spraying process constraints and motion smoothness among different algorithms.The gray shaded regions and vertical dashed lines indicate the turning regions of the S-shaped trajectory.

The degree to which various algorithms satisfy the process constraints and maintain the continuity of the spraying process is assessed using motion smoothness metrics, spray distance error, nozzle normal error, and spraying speed error. This study analyzes representative process data from an S-shaped spraying trajectory during the evaluation phase to track changes in these metrics during trajectory execution. The selected dataset has overall metric values close to the averages obtained from eight independent training runs, as shown in Figure 6.

The gray area indicates the turning regions of the S-shaped trajectory. As shown in Figure 6, all algorithms display different levels of peak fluctuation near normalized trajectory progress values of approximately 0.2, 0.4, 0.6, and 0.8. This indicates that the nozzle must simultaneously perform position adjustment, attitude coordination, and velocity switching when it moves from a lateral spraying segment to a line-change turning region. The performance metrics of the standard SAC show notable fluctuations. SAC+Composite Reward shows more evident reductions in spray distance error, nozzle normal error, spraying speed error, and the motion smoothness index, while SAC+PER reduces some local errors but has limited direct effects on spraying process constraints. The Improved SAC, on the other hand, shows reduced peak fluctuations and overall errors. As indicated in Table 3, Improved SAC produces a spray distance error of 23.6 ± 1.8 mm, a nozzle normal error of 5.9 ± 0.46°, a spraying speed error of 0.048 ± 0.004 m/s, and a motion smoothness index of 0.079 ± 0.006. The findings show that the proposed method can improve spraying process proxy indicators and motion continuity under the current digital twin environment and S-shaped trajectory conditions; however, these findings cannot yet be directly linked to an improvement in the actual sprayed-layer quality.

### 5.6. Visualization of Trajectory Execution in the Digital Twin Platform

**Figure 7:**
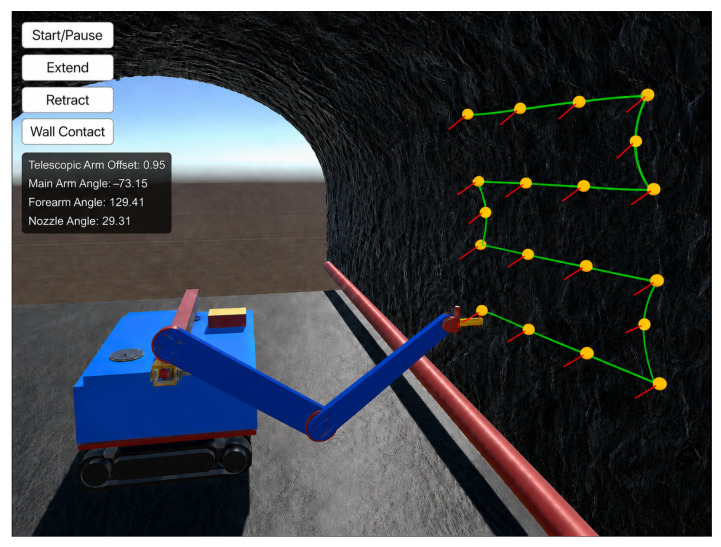
Virtual simulation scene of the layered reciprocating spraying trajectory. The green line represents the predefined S-shaped spraying path, the yellow circles denote the discrete sampling points along the trajectory, and the red segments indicate the nozzle orientation along the wall surface.

To further illustrate the trajectory execution process of the shotcrete robotic arm in the digital twin environment, this paper presents a visualization of the platform’s operating interface, as shown in Figure 7. The figure demonstrates the execution of a layered and reciprocating spraying trajectory by the robotic arm along the wall surface in a tunnel-like working environment. The green dashed line represents the predefined reference spraying path, while the left-side interface displays the platform’s basic control buttons and real-time joint status parameters, including telescopic arm displacement, main arm angle, forearm angle, and nozzle angle.

As shown in Figure 7, the digital twin platform is capable of displaying the robotic arm posture, working face environment, reference spraying trajectory, and nozzle direction within the same scene. This visualization intuitively reflects the spatial relationship between the spraying robotic arm and the working face and enables observation of the correspondence between the robotic arm posture and the predefined spraying path during trajectory execution.

## 6. Conclusions

By combining an enhanced SAC algorithm with double-critic TD-error-based PER and a composite reward function, this study proposed a digital-twin-based trajectory planning method for a 4-DOF spraying robotic arm. The two primary contributions of the proposed method are as follows. First, the PER mechanism improves the use of informative training samples by increasing the sampling probability of crucial transitions, such as trajectory turning areas, sudden spray distance changes, high-error states, and constraint-sensitive states. Second, the learned policy is more in line with the demands of the spraying task because the composite reward function integrates trajectory tracking, spray distance maintenance, nozzle normal alignment, spraying speed stability, safety constraints, and motion smoothness into the policy optimization objective.

The simulation results verify the effectiveness of the proposed method under the current digital twin environment and two-dimensional reference spraying trajectory. The Improved SAC reduces the trajectory tracking RMSE by 66.7%, from 24.0±2.0mm to 8.0±0.7mm. Furthermore, there is a reduction of 43.5%, 49.1%, 47.3%, and 48.7% in the spray distance error, nozzle normal error, spraying speed error, and motion smoothness index, respectively. These findings show that the proposed method improves trajectory tracking accuracy, spraying process proxy indicators, and motion smoothness in the simulated trajectory planning task.

However, the current study is still limited to simulation-based validation. Long-term actual spraying disturbances, numerous trajectory configurations, observation noise, control delays, and complex three-dimensional working surfaces have not yet been fully taken into account. Additionally, while sprayed-layer thickness uniformity, rebound rate, and construction efficiency have not been directly examined, the current evaluation mainly relies on trajectory errors and spraying process proxy indicators. Therefore, rather than direct evidence of consistent improvement in real underground spraying operations, the study’s outcomes should be interpreted as validation under the specified digital twin settings. Future work will focus on multi-scenario robustness tests, stronger baseline comparisons, physical testing without spraying material, and real-world spraying validation.

## Figures and Tables

**Table 1:** Joint variables and constraint parameters of the spraying robotic arm.

Joint	Variable	Joint Type	Unity Joint Type	Motion Range	Velocity Constraint
Telescopic arm	*d*	Prismatic joint	Prismatic	0≤d≤2.0m	$-0.10\;m/s\leq \dot{d}\leq 0.10\;m/s$
Upper arm	$\theta_{1}$	Revolute joint	Revolute	−150° $\leq \theta_{1}\leq 150$°	−20°$/s\leq {\dot{\theta }}_{1}\leq 20$°/s
Forearm	$\theta_{2}$	Revolute joint	Revolute	−150° $\leq \theta_{2}\leq 150$°	−20°$/s\leq {\dot{\theta }}_{2}\leq 20$°/s
Nozzle	$\theta_{3}$	Revolute joint	Revolute	−150° $\leq \theta_{3}\leq 150$°	−30°$/s\leq {\dot{\theta }}_{3}\leq 30$°/s

**Table 2:** Simulation experiment and algorithm parameter settings.

Category	Setting
Compared algorithms	SAC, SAC+PER, SAC+Composite Reward, Improved SAC
Ablation configurations	SAC is used as the baseline; SAC+PER only introduces PER; SAC+Composite Reward only introduces the composite reward; Improved SAC introduces both PER and the composite reward
Number of independent runs	8
Random seeds	2026, 2027, 2028, 2029, 2030, 2031, 2032, 2033
Training episodes	300 episodes
Number of trajectory sampling points	420
Smoothing window for convergence curves	9
State space dimension	19
Action space dimension	4
Network architecture	Actor: 19-256-256-4; Critic: 23-256-256-1
SAC parameters	Temperature coefficient α=0.20; discount factor γ=0.99; soft-update coefficient τ=0.005
Learning settings	Learning rate $=3.0\times 10^{-4}$; batch size =256; replay buffer capacity $=1.0\times 10^{6}$
PER parameters	Priority exponent η=0.6; importance sampling exponent β=0.4; priority constant $\varepsilon =1.0\times 10^{-4}$
Reward tracking weights	Position tracking weight $w_{p}=50$; nozzle normal error weight $w_{n}=1$
Spraying reward weights	Spray distance error weight $w_{d}=20$; spraying speed error weight $w_{v}=2$
Regularization reward weights	Control-amplitude penalty weight $w_{u}=0.01$; action-variation penalty weight $w_{\Delta u}=0.10$
Safety and completion reward weights	Obstacle-distance penalty weight $w_{o}=20$; trajectory-completion reward weight $w_{g}=10$
Safety penalty coefficients	Collision penalty coefficient $C_{\mathrm{col}}=20$; joint limit penalty coefficient $C_{\lim}=10$
Fair-comparison strategy	The same random seeds, network architecture, training episodes, trajectory sampling points, and basic SAC parameters are used for all compared algorithms; PER parameters and reward function parameters are used only in the corresponding variants
Primary evaluation metric	Trajectory tracking RMSE
Secondary evaluation metrics	Spray distance error, nozzle normal error, spraying speed error, and motion smoothness index
Normalization method	Convergence curves use min–max normalization; ablation metrics use SAC = 1 as the baseline

**Table 3:** Comprehensive performance descriptive statistics for various algorithms.

Algorithm	$E_{p}$/mm	$E_{d}$/mm	$E_{n}{/}^{\circ }$	$E_{v}$/(m/s)	$S_{u}$
SAC	24.0±2.0	41.8±3.1	11.6±0.88	0.091±0.007	0.154±0.011
SAC+PER	18.0±1.5	36.7±2.7	10.1±0.76	0.080±0.006	0.141±0.010
SAC+Composite Reward	13.0±1.1	28.9±2.1	7.5±0.57	0.060±0.005	0.103±0.008
Improved SAC	8.0±0.7	23.6±1.8	5.9±0.46	0.048±0.004	0.079±0.006

## Data Availability

The original contributions presented in this study are included in the article. Further inquiries can be directed to the corresponding author.

## Abbreviations

The following abbreviations are used in this manuscript:

SAC	Soft Actor–Critic
TD	Temporal Difference
DH	Denavit–Hartenberg
PER	Prioritized Experience Replay
DT	Digital Twin
RMSE	Root Mean Square Error
DoF	Degree of Freedom
